# Beyond the alarm: unveiling the multifaceted role of IL-33 in kidney disease

**DOI:** 10.3389/fimmu.2026.1887953

**Published:** 2026-08-14

**Authors:** Mei Yang, Suning Miao, Mingli Yu, Xiaoqing Fu, Xiaosong Qin

**Affiliations:** 1 Department of Laboratory Medicine, Shengjing Hospital of China Medical University, Shenyang, Liaoning, China; 2 Department of Pediatrics, Shengjing Hospital of China Medical University, Shenyang, Liaoning, China

**Keywords:** acute kidney injury, chronic kidney disease, IL-33, immunomodulation, ST2 receptor

## Abstract

Interleukin-33 (IL-33) has long been categorized as a prototypical alarmin released upon cellular injury to initiate Th2-skewed immune responses. However, accumulating evidence from diverse kidney disease models and patient cohorts has unveiled a far more complex and contextually governed functionality. This review synthesizes recent advances to reframe the understanding of IL-33, positioning it not merely as a passive danger signal, but as an active and versatile rheostat of renal pathophysiology. The net effect of IL-33/ST2 signaling, whether tissue-protective or pro-inflammatory and fibrotic, is critically dictated by the disease microenvironment, encompassing the nature of the initial insult (e.g., ischemia–reperfusion, nephrotoxic agents, or autoimmune complexes), the phase of disease (acute versus chronic), and the local immune–parenchymal cell network. We delineate how the IL-33/ST2 axis differentially orchestrates key immune effectors, including group 2 innate lymphoid cells (ILC2s), macrophage polarization states, and T cell subsets with particular emphasis on regulatory T cells (Tregs). These interactions in turn shape divergent clinical outcomes, ranging from effective tissue repair and functional recovery to relentless inflammation and progressive fibrosis. By integrating findings across acute kidney injury, diabetic nephropathy, lupus nephritis, and IgA nephropathy, this review underscores the dual, context-dependent nature of IL-33 signaling. We further discuss its emerging potential as a stratified biomarker and a modifiable therapeutic target, and advocate for future strategies that precisely calibrate IL-33/ST2 activity according to specific pathological contexts, with the goal of achieving optimized and durable renal outcomes.

## 1 Introduction

Kidney diseases represent a public health challenge on a global scale, with acute kidney injury (AKI) and chronic kidney disease (CKD) emerging as priority areas for prevention and control due to their high incidence, poor prognosis, and substantial healthcare burden (1–3). AKI is defined as a clinical condition marked by a swift reduction in kidney function, caused by multiple factors (4). Severe cases of AKI may progress to CKD or even result in irreversible renal failure (4, 5). According to the underlying pathological mechanisms, CKD is categorized into primary glomerular diseases and secondary kidney diseases. Primary glomerular diseases are the predominant cause of glomerulopathy (6), with idiopathic membranous nephropathy (IMN) being the most prevalent primary nephropathy, followed by IgA nephropathy (6–9). Among secondary nephropathies, diabetic nephropathy is the most prevalent, succeeded by lupus nephritis (6).

In the context of renal injury and repair, inflammation serves as a critical intermediary (10, 11). Beyond infection, several factors contribute to renal inflammation, including ischemia-reperfusion (12), immune complex formation or deposition (13–16), and the activation of pro-inflammatory pathways (17–20). Notably, primary damage to renal tissue can lead to the generation of cytokines and chemokines, serving as mediators of inflammation (10, 17). Recent investigations into the key regulatory factors of renal inflammation and repair have highlighted the IL-33/ST2 signaling axis as a pivotal area of investigation (21). Interleukin-33 (IL-33), belonging to the IL-1 cytokine superfamily, is abundantly enriched within the nuclei of human epithelial, endothelial, and fibroblast lineages. It is also detectable in the nuclei of various mouse tissues, including epithelial cells, lymphoid organs, brain, and embryonic tissues (22–24). Its role in human diseases is diverse (25). The receptor for IL-33, known as suppression of tumorigenicity 2 (ST2), is expressed by a wide range of immune cells, including group 2 innate lymphoid cells (ILC2s), Th2 cells, mast cells, eosinophils, basophils, dendritic cells, regulatory T cells (Tregs), NK cells, invariant natural killer T (iNKT) cells, M2-polarized macrophages, and neutrophils (26). ST2 is present in both a transmembrane form, known as the transmembrane receptor ST2 (ST2L), and a soluble excretory form, referred to as soluble ST2 (sST2) (26, 27). Within the nucleus, IL-33 associates with chromatin (28). Upon tissue damage or necrosis of cells, IL-33 is secreted into the extracellular milieu, where it engages in an autocrine or paracrine fashion by binding to its receptor, ST2 (26, 29). This interaction constitutes the IL-33/ST2 signaling pathway, which is vital for modulating a range of pathophysiological processes, including inflammatory responses, angiogenesis, and apoptosis (26, 30, 31). The engagement of IL-33 with its cognate receptor complex, comprised of ST2 and interleukin-1 receptor accessory protein (IL-1RAcP), initiates a cascade of intracellular signaling events. This heterodimeric complex serves as a scaffold for the assembly of key adaptor proteins, notably myeloid differentiation primary response 88 (MyD88), interleukin-1 receptor-associated kinase 1 and 4 (IRAK1/4), as well as tumor necrosis factor receptor-associated factor 6 (TRAF6). The subsequent recruitment of these molecules triggers the activation of the mitogen-activated protein kinase (MAPK) module. In turn, this leads to the stimulation of transcription factors, including nuclear factor kappa B (NF-κB) and activator protein 1 (AP-1), which ultimately upregulate the transcriptional output of genes governing inflammatory responses, cellular viability, and fibrotic processes (32, 33).

Recent studies have identified an increasingly significant role for IL-33 in kidney diseases. In the context of AKI, IL-33 predominantly exhibits a pro-injury effect, exacerbating renal inflammation and fibrosis, while also contributing to tissue repair. Similarly, in CKD, the IL-33/ST2 signaling pathway demonstrates dual roles, with its protective and pathogenic effects contingent upon the specific type of disease and the duration of signal transduction (21). By systematically reviewing the current evidence on the IL-33/ST2 axis across different renal pathologies, this article highlights its context-dependent regulatory functions and provides a foundation for better understanding the mechanistic complexity of this pathway in kidney diseases.

## 2 The role of IL-33 in acute kidney injury

**Figure 1:**
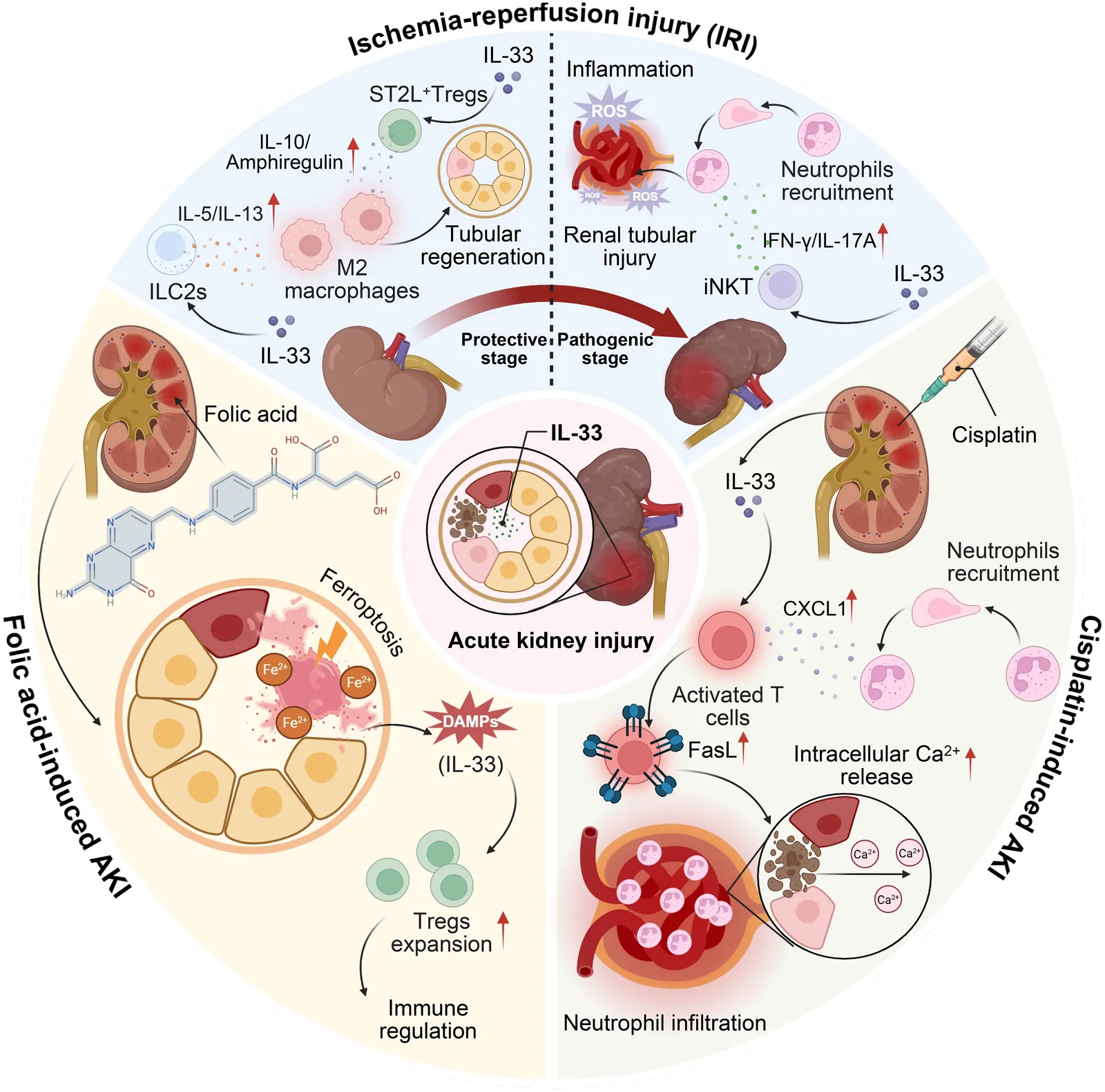
Role of IL-33 in acute kidney injury. IL-33 exerts a dual regulatory role in three distinct models of AKI: IRI, cisplatin nephrotoxicity, and folic acid-induced AKI. During the early stage of IRI, IL-33 activates iNKT cells to secrete IFN-γ and IL-17A, which mediate neutrophil recruitment and reactive oxygen species (ROS) burst, aggravating tubular injury. In contrast, during the late stage, IL-33 acts on ILC2s and ST2^+^Tregs to induce M2 macrophage polarization and the secretion of anti-inflammatory factors and amphiregulin, thereby promoting renal tubular regeneration and inflammation resolution. In cisplatin-induced AKI, IL-33 upregulates the chemokine CXCL1 via CD4^+^T lymphocytes, mediating neutrophil infiltration and exacerbating renal injury. In folic acid-induced AKI, ferroptosis of renal tubular epithelial cells releases IL-33, which in turn promotes Treg expansion and modulates M1/M2 macrophage polarization, initiating local immune responses.

The incidence of AKI predominantly affects epithelial cells, endothelial cells, and other parenchymal cells within specific renal regions (34). Following tissue or cellular damage, an inflammatory response is promptly initiated, mediated by both resident and infiltrating immune cells, including neutrophils and macrophages (34, 35). Clinically, AKI is characterized by a high risk of recurrence and prolonged injury duration, with the potential to progress to advanced CKD and end-stage kidney disease (ESKD) (36). In the context of AKI, the expression of IL-33, a critical regulatory factor in inflammation, is elevated, and its functions are heterogeneous (37–41). To elucidate its mechanism, several AKI models have been established to explore pathogenesis and assess therapeutic strategies (42), revealing that IL-33 plays diverse roles across different models. The distinct functions of IL-33 in ischemic-reperfusion injury (IRI), cisplatin-, and folic acid-induced AKI are summarized in **Figure 1**.

### 2.1 Model of ischemia-reperfusion injury

In the context of IRI, IL-33 has been demonstrated to encourage the expansion of ILC2s, M2 macrophages, and Tregs, thereby initiating Th2 immune responses and concurrently suppressing the production of proinflammatory factors, which collectively confer a protective effect on renal tissues (38). Furthermore, numerous studies have identified a pro-fibrotic role of IL-33 in the pathophysiology of IRI (43, 44). These findings suggest that, beyond its reparative role in acute kidney injury models, IL-33 also contributes to kidney disease pathogenesis. Following IRI-induced damage, IL-33 levels increase in the kidney, facilitating the accumulation of fibroblasts from bone marrow, the generation of myofibroblasts, as well as the recruitment of macrophages and T cells, ultimately leading to renal injury and fibrosis (45). In the initial stages of IRI, IL-33 directly impacts renal tubular epithelial cells, causing renal tubular necrosis and impaired renal function (29). As the disease progresses, IL-33, under the synergistic influence of IL-12, can recognize the ST2 receptor on the surface of iNKT cells. This engagement promotes the mobilization and stimulation of iNKT cells, prompting them to secrete interferon-gamma (IFN-γ) and IL-17A, which subsequently act on neutrophils. This process enhances the recruitment of neutrophils to the kidney, thereby exacerbating IRI (29, 46).

The regulation of IL-33 expression involves complex epigenetic mechanisms. The chromatin remodeling protein brahma-related gene-1 (BRG1) has been shown to mediate IL-33 expression in a hypoxia-inducible factor-1α (HIF-1α)-dependent manner, involving histone-modifying proteins such as p300, ASH2, and KDM3A, thereby promoting renal injury and fibrosis induced by IRI (43). However, it is important to note that BRG1 is a broad regulator of the inflammatory response; it also promotes the transcription of other proinflammatory cytokines, including TNF-α, IFN-γ, and MCP-1, in the kidney following ischemic injury (47). Therefore, the protective effects observed upon BRG1 deletion are likely attributable to the suppression of a panel of proinflammatory mediators, rather than solely the downregulation of IL-33.The IL-33/ST2 axis also plays a critical role in modulating the function of Tregs during renal IRI. Emerging evidence indicates that ST2 expression on Tregs is essential for their immunosuppressive and tissue-reparative functions (48). Treg-specific deletion of ST2 has been shown to exacerbate kidney injury, inflammation, and fibrosis in murine models of IRI, underscoring the protective role of the ST2^+^Treg subset in the resolution of renal injury (48, 49). Mechanistically, ST2^+^Tregs are enriched for the expression of reparative factors, including amphiregulin (AREG), which contributes to the preservation of tubular epithelial cell viability and the suppression of fibrogenesis (50). Collectively, these findings highlight the dual nature of the IL-33/ST2 axis, which can both promote and resolve renal IRI depending on the cellular context and the stage of injury.

### 2.2 Models induced by cisplatin and folic acid

In the cisplatin-induced AKI model, the role of IL-33 is context-dependent. In an acute, high-dose model, IL-33 facilitates the production of the chemokine CXCL1 by CD4^+^ T cells via the ST2 receptor, which subsequently leads to neutrophil recruitment or directly induces cellular damage through the release of intracellular Ca^2+^ and the expression of FasL, thereby exacerbating renal injury (51). However, in a chronic, low-dose model of cisplatin-induced AKI in tumor-bearing mice, genetic deficiency of IL-33 did not provide functional or histological protection, suggesting that the IL-33/CD4 T-cell/CXCL1 axis may not be a major mediator in this more clinically relevant setting (52).

In the folic acid (FA)-induced AKI model, IL-33 functions as a damage-associated molecular pattern (DAMP) and can be released extracellularly in a ferroptosis-dependent manner, thereby exerting a pro-inflammatory effect (53). However, the role of IL-33 in this model is not unidirectional. While its upregulation is associated with injury, studies have shown that genetic deletion of the necroptosis regulator receptor-interacting protein kinase 3 (RIPK3) did not attenuate IL-33 expression but did reduce other proinflammatory cytokines. This has led to the hypothesis that IL-33, in addition to being an early immune activator, may also play a role in immune regulation, potentially via Treg cells, to limit excessive inflammation and facilitate tissue repair (53).

## 3 The role of IL-33 in chronic kidney disease

Research indicates that AKI significantly elevates the risk of developing CKD, which is characterized by organ fibrosis (54). In cases of CKD patients, there is a marked increase in the levels of IL-33 and its soluble decoy receptor sST2 (55). Notably, sST2 competes with the membrane-bound receptor ST2L for IL-33 binding, thereby functioning as a negative regulator of IL-33/ST2 signaling, which may modulate the net biological activity of IL-33 in the CKD setting. In the context of CKD, IL-33 interacts with the ST2L receptor, subsequently activating the NF-κB and MAPK signaling pathways via the classical MyD88-IRAK-TRAF cascade. This process aids in activating ILC2s, enhances the secretion of IL-5 and IL-13, and sustains the polarized state of M2 macrophages (56). M2 macrophages are capable of releasing mediators such as IL-10, arginase-1, and Areg, which contribute to anti-inflammatory effects and tissue repair (57, 58). The IL-33/ST2 signaling axis has been linked to regulation of the transforming growth factor-beta (TGF-β) pathway, thereby driving renal epithelial-mesenchymal transition (EMT) and ultimately contributing to the progression of renal fibrosis (59). Research indicates that M2 macrophages may not only mitigate renal inflammation but also facilitate renal fibrosis through the secretion of TGF-β (58, 60, 61). This underscores the heterogeneous role of IL-33 in the pathophysiology of CKD.

### 3.1 The role of IL-33 in lupus nephritis

Research indicates that sST2 and ST2L levels are markedly elevated in individuals with lupus nephritis (LN) (62). IL-33 interacts with ST2, facilitating both the clinical and pathological progression of LN (21). Systemic lupus erythematosus (SLE), an autoimmune disorder, frequently impacts the kidneys, leading to LN (63). The IL-33/ST2 signaling pathway has been demonstrated to stimulate plasmacytoid dendritic cells (pDCs) to produce substantial quantities of interferon-alpha (IFN-α) by altering neutrophil extracellular traps (NETs). This process exacerbates autoimmune inflammation and contributes to end-organ damage in SLE. Consequently, IL-33 potentially contributes to the neutrophil/NETs-IL-33/IFN-α pathway, further advancing renal inflammation (64, 65). By contrast, serum sST2 levels were found to be significantly elevated in patients with active SLE compared with those with inactive disease and correlated with SLEDAI, anti-dsDNA antibody levels, and complement consumption, whereas circulating IL-33 levels showed no consistent association with disease activity (66). Clinical data show a positive correlation between IL-33/ST2 axis activation and LN severity, but mechanistic experiments establish a unidirectional cascade: nucleotide-binding oligomerization domain-like receptor family pyrin domain containing 3 (NLRP3) drives IL-33, while IL-33 knockdown does not affect NLRP3, excluding reciprocal feedback. Thus, the NLRP3/IL-33/ST2 axis represents linear forward signaling from NLRP3 to IL-33, not bidirectional crosstalk (62).

Nevertheless, a growing body of evidence points to a protective function for IL-33 in certain pathological settings. For instance, in the NZB/W F1 murine model – a first-generation cross between New Zealand Black and New Zealand White strains that exhibits a pronounced susceptibility to lupus-like autoimmunity – early administration of recombinant IL-33 has been shown to markedly ameliorate proteinuria, prolong overall survival, and attenuate renal injury. This is achieved by enhancing the production of anti-dsDNA IgM antibodies, increasing the population of IL-10+ regulatory B cells, and inducing the expression of genes associated with M2 macrophages (67). Conversely, in the MRL-lpr model and the imiquimod (IMQ)-induced model, research indicates that ILC2s during autoimmune kidney disease diminish the increased expression of integrin α4β7 in healthy kidneys via Toll-like receptor 7/9 (TLR7/9) signaling. This process leads to the downregulation of cytokines, such as the regulatory protein Areg, thereby promoting renal inflammation. However, treatment with IL-33 has been shown to elevate the levels of α4β7 and Areg, thereby alleviating renal inflammation (68).

### 3.2 The role of IL-33 in IgA nephropathy

B cell activating factor (BAFF), which is part of the tumor necrosis factor (TNF) superfamily, is key to controlling the maturation and differentiation of B lymphocytes. This cytokine exerts a profound influence on both the proliferative expansion and developmental progression of the B cell compartment (69, 70). In BAFF-transgenic (BAFF-Tg) mice, excessive expression of IL-33 can elevate BAFF levels, leading to IgA nephropathy (IgAN) which involves circulating immune complexes and the deposition of immunoglobulins (71, 72). Furthermore, administration of IL-33 may enhance IgA production by expanding ILC2s, thereby inducing immune complex deposition and exacerbating renal lesions (71–73). Additionally, elevated concentrations of BAFF can stimulate B cells to produce IgA immune complexes, which deposit in the glomeruli and interact with mesangial cells. Activated mesangial cells can recruit inflammatory cells, such as macrophages, to further promote glomerular damage and also cause injury to podocytes (73, 74).

It is important to note that while BAFF-Tg mice develop a form of IgAN involving circulating immune complexes and immunoglobulin deposition, this model primarily reflects pathology driven by BAFF overexpression and subsequent polyclonal B-cell hyperplasia (71). This differs from the current understanding of human IgAN pathogenesis, which is characterized by the generation of autoantibodies, specifically against aberrantly galactosylated IgA1, leading to the formation and glomerular deposition of IgA1-containing immune complexes (73).

However, it must be highlighted that in the referenced studies, IL-33 administration alone did not cause the disease. The inflammatory environment and cell death resulting from BAFF overexpression could themselves contribute to increased IL-33 levels (71). Furthermore, direct evidence for the exacerbation of specifically agalactosylated IgA production or the generation of anti-IgA antibodies following IL-33 treatment was not reported (72). In the context of human IgAN, elevated serum BAFF levels have been correlated with disease activity, suggesting a potential role in the amplification of the autoimmune response and subsequent renal injury (73–75). Single nucleotide polymorphism (SNP) analysis has indicated that increased levels of sST2 are associated with IgA glomerulonephritis (55, 76, 77). Previous research has indicated that, upon investigating the upstream factors of ILC2s, IL-33 levels do not differ significantly between individuals with IgAN and those who are healthy. However, serum sST2 levels in IgAN patients exhibit a positive correlation with the severity of the disease (76). The findings suggest that sST2 might have a function in the negative modulation of IL-33 by acting as a decoy receptor, potentially sequestering IL-33 and attenuating its signaling, rather than indicating increased IL-33 bioactivity in IgAN pathogenesis.

### 3.3 The role of IL-33 in diabetic nephropathy

Diabetic nephropathy (DN) is a major complication associated with diabetes mellitus (78). The cytokine IL-33 has been implicated in the pathophysiology of diabetes (79), with elevated concentrations of IL-33 and its receptor ST2 detected in DN (80). However, it is important to note that a review of observational studies found that while IL-33 levels did not consistently correlate with disease severity, soluble ST2 (sST2) levels did show a positive correlation with disease progression. sST2 functions as a decoy receptor that binds IL-33 and prevents its signaling through the membrane-bound ST2 receptor, suggesting that elevated sST2 in DN may reflect systemic inflammation and consequent attenuation of IL-33’s beneficial effects (80). Empirical studies have demonstrated a progressive increase in IL-33 levels correlating with the progression of DN, with markedly higher levels detected in patients with advanced-stage DN compared to those in the early stages (81). Furthermore, IL-33 has been linked to cellular senescence in DN, exhibiting positive correlations with ST2 and the senescence-associated protein p16 in DN patients. Experimental evidence indicates that recombinant IL-33 (rIL-33) exacerbates senescence-related phenotypes in DN mouse models, whereas treatment with neutralizing anti-IL-33 antibodies (αIL-33) mitigates cellular senescence in these models (81). These observations indicate that reducing IL-33 expression could cause a decrease in p16 levels, a key player in cellular senescence regulation (82)—thereby inhibiting cellular senescence and alleviating cellular damage in DN (81). The generation of IL-33 and ST2 knockout mice (IL-33−/− or ST2−/−) has demonstrated that the absence of these genes exacerbates glomerular damage, increases tubulointerstitial injury scores, and raises levels of Kidney Injury Molecule-1 (KIM-1). Conversely, a reduction in IL-33 signaling, either through exogenous administration or endogenous mechanisms, in αIL-33-treated mice and IL-33+/− and ST2+/− heterozygous mice, leads to an improvement in renal injury associated with DN (81). These paradoxical findings suggest a complex, non-linear (biphasic) relationship between IL-33 signaling and renal outcomes: complete loss of IL-33/ST2 signaling is detrimental, while partial reduction may be beneficial, and excessive IL-33 (as seen with recombinant IL-33 administration) is also harmful (81).

The protective role of IL-33 in diabetes and its complications is supported by multiple independent lines of evidence. In the BTBR.Cg-Lep^ob^/^ob^ mouse model of type 2 diabetic nephropathy, treatment with IL233—a novel bifunctional cytokine bearing both IL-2 and IL-33 activities—reduced hyperglycemia, attenuated albuminuria, and preserved renal structure and function. This protection correlated with increased accumulation of Tregs, ILC2s, alternatively activated M2, and eosinophils in adipose tissue, along with skewing toward Th2 immune responses (83). In the multiple low-dose streptozotocin (MLD-STZ) model of type 1 diabetes, exogenous IL-33 treatment completely prevented the development of hyperglycemia and insulitis in C57BL/6 mice, and attenuated insulitis in prediabetic NOD mice. These protective effects were accompanied by increased numbers of CD4^+^IL-5^+^, CD4^+^IL-13^+^, and CD4^+^Foxp3^+^ST2^+^ regulatory T cells in pancreatic lymph nodes and islets (84). Similarly, administration of IL-33 to prediabetic NOD mice decreased the incidence and delayed the onset of autoimmune diabetes, an effect associated with increased ST2^+^GATA3^+^ Treg cells and activation of p38/Erk MAPK pathways (85). In obese mouse models, IL-33 treatment reduced visceral adiposity, improved glucose tolerance and insulin sensitivity, and promoted the accumulation of Th2 cells and M2 macrophages in adipose tissue (86). Moreover, IL-33-activated islet-resident ILC2s promote insulin secretion through myeloid cell retinoic acid production (87), and IL-33 promotes adipocyte lipolysis via β-adrenergic receptor/cAMP/PKA/HSL signaling (88). Notably, IL-33 expression in human subcutaneous adipose tissue is elevated in insulin resistance and type 2 diabetes, and IL-33 treatment inhibits adipocyte glucose uptake (89). Furthermore, ST2 deficiency attenuates renal ischemia-reperfusion injury in euglycemic mice but not in hyperglycemic mice, and plasma sST2 levels are elevated in type 2 diabetes patients, suggesting that the IL-33/ST2 pathway exerts differential effects depending on the glycemic environment (90). Moreover, IL-33 markedly suppresses the transcriptional expression of genes linked to PERK and IRE1 signaling, as well as endoplasmic reticulum (ER) stress-associated markers including GRP78, CHOP, and caspase-12, thus attenuating diabetic kidney injury (91).

## 4 Roles of IL-33 in pulmonary, cardiovascular, and hepatic diseases and comparison with renal mechanisms

As an alarmin released upon tissue injury, IL-33 exerts context-dependent dual functions across multiple organs. Signaling converges on the IL-33/ST2 axis, which initiates type 2 immunity and repair during the acute phase, whereas chronic stimulation drives fibrosis and remodeling. Outcomes are shaped by cellular sources, tissue microenvironment, and disease time window (92). In the lung, IL-33 from airway epithelium activates ILC2s and Th2 cells, releasing IL-4/IL-5/IL-13, leading to eosinophilic inflammation and airway remodeling in asthma; anti-IL-33 therapy attenuates this, though bypass pathways may limit efficacy (93). In COVID-19, IL-33 shifts from acute pro-inflammatory to late pro-fibrotic (94). In the cardiovascular system, IL-33 from endothelial cells and macrophages shows species-specific effects: it protects against atherosclerosis and myocardial injury in murine models via M2 polarization, but promotes endothelial inflammation and thrombosis in human settings; sST2 serves as a heart failure prognostic marker (95, 96). In the liver, acute IL-33 release expands ILC2s, promotes M2 macrophages, and facilitates hepatocyte proliferation via the gut–liver axis, supporting regeneration and fibrosis resolution (97–99); chronic activation of the IL-33/ILC2/Th2 axis induces IL-13, driving hepatic stellate cell activation and collagen deposition, accelerating fibrosis (100, 101). Serum IL-33 correlates with fibrosis stage, suggesting biomarker utility (101).

**Figure 2:**
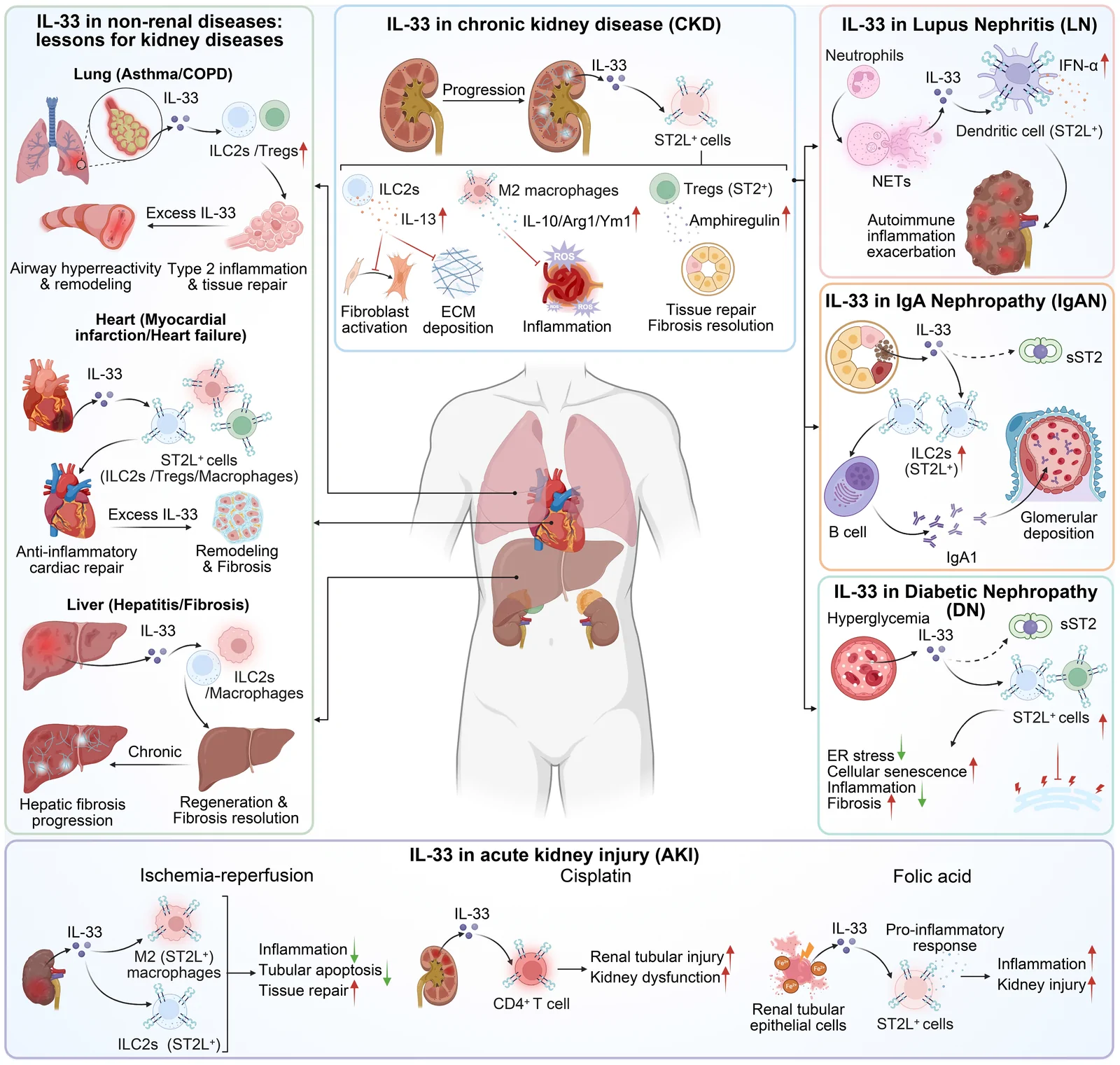
Mechanisms of IL-33 action in extrarenal diseases and various kidney diseases. IL-33 exerts dual regulatory roles in tissue inflammation, fibrosis, and damage repair by acting through the ST2 receptor on various immune cell subsets, including ILC2s, Tregs, and macrophages. Its broad involvement in pathological progression and tissue repair in non-renal diseases (lung, heart, and liver) offers important lessons for understanding its role in kidney diseases. In kidney diseases, the effects of IL-33 are highly heterogeneous. It regulates the balance between fibrosis and repair in CKD, drives the pathological progression of LN, IgAN, and DN, and exhibits distinct roles across different AKI models: it is predominantly reparative in ischemia-reperfusion injury, whereas in cisplatin- and folic acid-induced models, it aggravates injury through CD4^+^ T cell and renal tubular epithelial cell-mediated pro-inflammatory responses, respectively. Furthermore, sST2 acts as a decoy receptor, exerting a negative regulatory effect within this signaling axis.

In the kidney, IL-33 from tubular epithelial cells, podocytes, and fibroblasts exerts transient protective effects during acute injury but drives TGF-β/CTGF-mediated interstitial fibrosis under chronic stimulation; sST2 levels correlate with disease progression (92, 102) Across organs, the IL-33/ST2 axis mediates type 2 immune imbalance that promotes fibrosis, yet each organ displays distinct pathology: eosinophilic airway inflammation in lung, endothelial/coagulation disorders in cardiovascular system, stellate cell-driven fibrogenesis in liver, and interstitial fibrosis in kidney (92). This comparison confirms that IL-33 effects are tissue- and stage-dependent, supporting precise targeting of this pathway and reinforcing the context-dependent framework of this review (92). The organ-specific actions of IL-33 in pulmonary, cardiovascular, and hepatic diseases, alongside its distinct mechanistic pathways in CKD, LN, IgAN, DN, and AKI, are comprehensively summarized and contrasted in **Figure 2**.

## 5 The mechanisms of action of IL-33 in renal diseases

**Figure 3:**
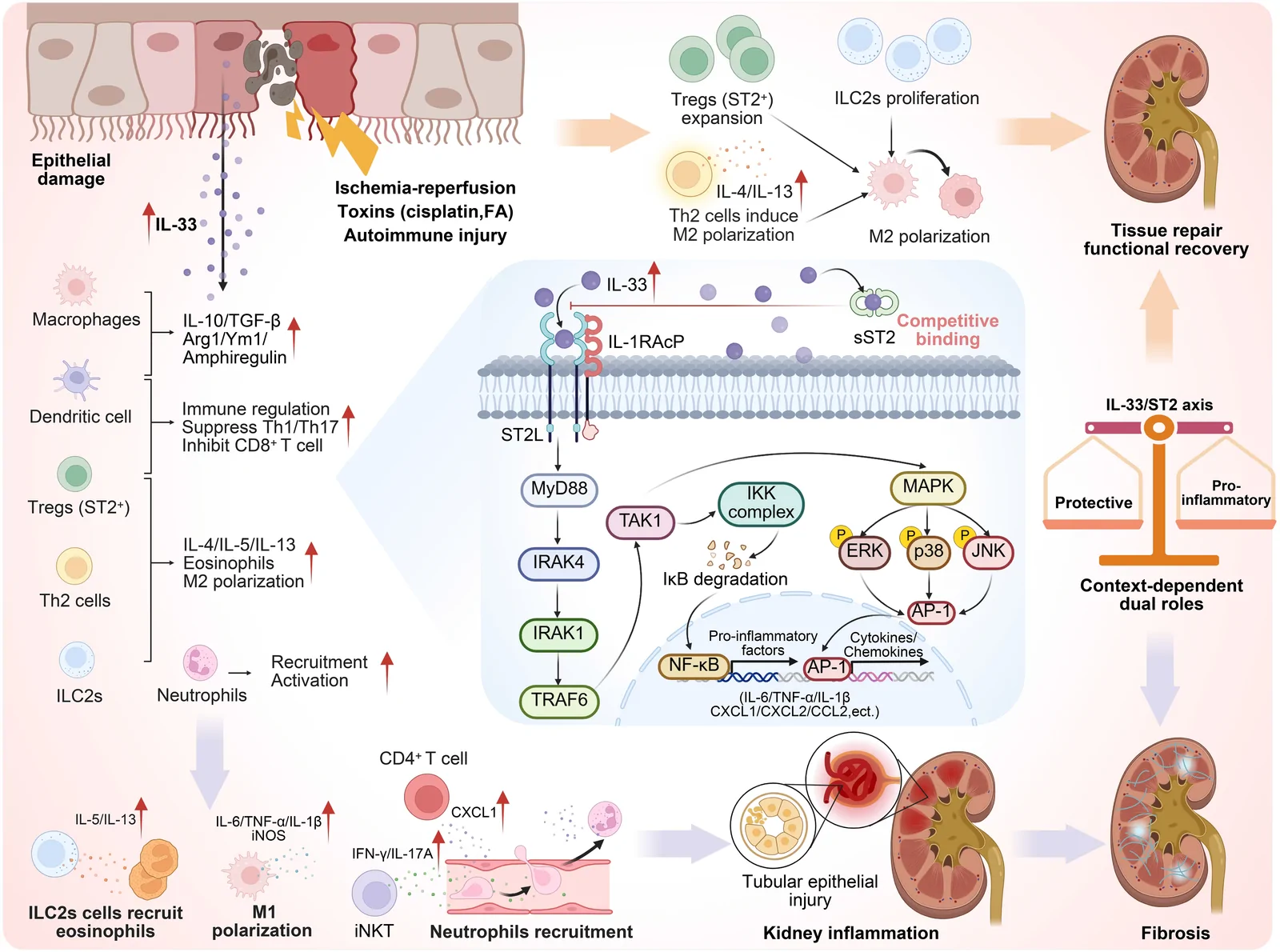
The dual role of the IL-33/ST2 signaling axis in kidney diseases. Renal injury induced by ischemia-reperfusion, nephrotoxic agents, and autoimmunity triggers the release of IL-33, which binds to the ST2L/IL-1RAcP receptor complex and activates the NF-κB and MAPK pathways through the MyD88-IRAK4/1-TRAF6-TAK1 cascade. sST2 acts as a decoy receptor that competitively binds IL-33, thereby negatively regulating the activation of this axis. The IL-33/ST2 axis exerts context-dependent dual effects. On one hand, it mediates anti-inflammatory repair and promotes renal functional recovery by regulating Treg expansion, ILC2 proliferation, and Th2 cell-induced M2 macrophage polarization. On the other hand, it exacerbates tubular epithelial injury, renal inflammation, and ultimately leads to fibrosis by inducing M1 macrophage polarization, neutrophil infiltration, and activation of pro-inflammatory T cells (CD4^+^ T cells and iNKT cells).

In various renal pathologies, IL-33 exhibits differential effects on disease onset, progression, and renal outcomes through a range of mechanisms, ultimately demonstrating both pro-injury and pro-repair roles (refer to **Table 1**). The principal mechanism of IL-33 involves its binding to the ST2 receptor, thereby forming a signaling pathway that recruits adaptor molecules such as MyD88 and IRAK1. This interaction activates the MAPK signaling cascade, subsequently triggering transcription factors such as NF-ĸB and AP-1, which in turn drive the expression of genes associated with inflammation, cell survival, and fibrosis (32). Furthermore, IL-33 facilitates the expansion of ILC2s and enhances their secretion of IL-5 and IL-13 via the JAK3-STAT5 signaling pathway. In addition, IL-33 is capable of initiating innate immune responses, promoting the release of Th2 cytokines, and activating macrophages (103). **Figure 3** summarizes the functional pathways mediated by IL-33.

**Table 1:** Mechanisms underlying IL-33 function.

Core mechanisms	The mechanisms of action of IL-33	Role of IL-33
ILC2s	Expansion of ILC2s	Mitigate renal injury and suppress fibrosis (37, 38, 40, 106, 107)
	Induce the secretion of IL-5 and IL-13 by ILC2s	Facilitate tissue regeneration (56, 108)
		Aggravate pathological damage (109)
Macrophages	Activate type 2-related effector cells, induce the release of Th2 cytokines, and stimulate macrophage activation	Pro-inflammatory and pro-fibrotic (103)
	Promote the accumulation of M2 macrophages	Improve prognosis and alleviate injury (38, 67, 108)
	Induce the phenotypic differentiation of monocytes into alternatively activated M2 macrophages	Promote epithelial regeneration and tissue repair (56)
	M2 macrophage polarization	Aggravate damage (109)
T cells	CD4^+^ T cell-dependent	Aggravate damage (51)
	Target iNKT cells	Alleviate fibrosis (29, 110)
Tregs	ST2-GATA3-Foxp3 loop activation, metabolic/epigenetic remodeling, and IL-2 modulation.	Treg homeostasis, immunosuppression, and repair initiation (32, 111–113)
	AREG induction, ILC2 synergy, and Arg1^+^ M2 generation via crosstalk.	Tissue regeneration and early AKI-to-CKD blockade (32, 48, 104, 114, 115)
	Sustained ILC2/Th2 activation, IL-13 excess, and sST2/IL-33 ratio regulation.	Time-dependent double-edged effects (fibrosis risk) and prognosis monitoring (48, 50, 105, 111)
Signaling pathway	Forms the IL-33/ST2 signaling pathway by binding to ST2, recruits adaptor molecules such as MyD88 and IRAK1, activates the MAPK signaling pathway, and thereby triggers transcription factors including NF-ĸB and AP-1	Pro-inflammatory and pro-fibrotic (26)
	TGF-β signaling pathway	Pro-fibrotic (59)
	EGFR signaling pathway	Facilitate epithelial regeneration while suppressing excessive inflammatory responses (116)
	MAPK signaling pathway	Pro-inflammatory and pro-fibrotic (32, 117)
	Independent of the ST2 signaling pathway	Inhibit the progression of fibrosis (118)
Immune regulation	Initiate the innate immune response and facilitate the production of IFN-γ and IL-17A	Aggravate renal damage (29)
	Promote the production of anti-dsDNA IgM antibodies and facilitate the expansion of IL-10^+^ regulatory B cells	Mitigate renal injury (67)
Endoplasmic reticulum	Reduce the expression levels of PERK and IRE1	Suppress cellular apoptosis and mitigate ER stress (105)
	Inhibit the activation of GPR78, CHOP, PERK, and IRE1 in glomerular endothelial cells	Suppress cellular apoptosis and mitigate ER stress (91)
Interact with other molecules	Enhance the expression of BAFF, thereby promoting the maturation and differentiation of B cells, and facilitating the formation of immune complexes	Pro-inflammatory (71–73)
	Released extracellularly in a manner dependent on ferroptosis	Pro-inflammatory (53)
	Engage with histone-modifying proteins, the NLRP3 inflammasome, and various other molecules, thereby modulating its own expression and subsequent inflammatory responses	Pro-inflammatory (43, 45, 62)

IL-33 activates tissue-resident ST2^+^Tregs (CD4^+^Foxp3^+^GATA3^+^), forming an ST2-GATA3-Foxp3 positive feedback loop that stabilizes Foxp3, promotes Treg proliferation, and enhances immunosuppressive capacity, while also maintaining Treg homeostasis via metabolic and epigenetic remodeling (32). IL-33 induces ST2^+^Tregs to secrete AREG, a core mediator of renal tissue repair; Treg-specific ST2 or AREG deletion exacerbates ischemia–reperfusion injury, whereas exogenous AREG restores protection (32, 48). IL-33 also cooperates with Tregs and ILC2s to promote Arg1^+^M2 macrophage generation, amplifying immune tolerance (104). The IL-33/ST2-Treg axis exerts time-window-dependent dual effects: early moderate activation suppresses acute inflammation and initiates AREG-mediated repair, blocking AKI-to-CKD transition, whereas sustained overactivation drives persistent ILC2/Th2 and IL-13 release, promoting long-term fibrosis (48, 50, 105). Clinically, the sST2/IL-33 ratio may serve as a prognostic biomarker and therapeutic monitor (32, 50). Details of the aforementioned mechanisms are presented in **Table 1** and **Figure 3**.

## 6 Clinical applications of IL-33 in renal diseases

### 6.1 As a diagnostic assessment marker

Research has demonstrated that there are notable differences in IL-33 levels between individuals with kidney diseases and healthy controls, indicating that IL-33 expression may correlate with disease severity. However, a systematic review of observational studies on the IL-33/ST2 axis in diabetic kidney disease found that while IL-33 levels did not consistently correlate with disease progression, sST2 showed a stronger and more consistent association with deteriorating kidney function, suggesting that sST2 may have greater utility as a diagnostic or prognostic biomarker (80). Elevated sST2 levels in CKD and diabetic kidney disease correlated with disease severity, possibly reflecting systemic inflammation and attenuation of IL-33’s protective effects via decoy receptor activity (80). These findings indicate that measuring components of the IL-33/ST2 pathway in plasma could facilitate the assessment of disease status during kidney disease diagnosis, thereby offering a non-invasive alternative to renal biopsy. Recent studies have identified molecules linked to the progression of CKD through transcriptome analysis and the development of risk models. Construction of risk prediction models relies on these molecules, which span multiple functional classes. Each class exerts an influence on unique biological signaling routes, transcriptional control factors, injury-associated indicators, and specific cellular populations. Consequently, they modulate the trajectory and ultimate resolution of the disease in distinct manners (119–121). Given the complex relationship between IL-33, its decoy receptor sST2, and disease progression, integrating the biological properties of this pathway with multi-omics analysis and machine learning algorithms could improve our comprehension of the molecular mechanisms underlying kidney diseases and enhance prediction of patient prognosis. However, further validation of these approaches is necessary.

### 6.2 As a therapeutic target: evidence and challenges

In recent years, IL-33 has gained attention as a potential treatment target for kidney diseases, with related research progressing to the clinical translation and validation stage. A representative example is the phase 2b FRONTIER-1 trial conducted in patients with type 2 diabetes-associated CKD. In this study, the anti-IL-33 monoclonal antibody tozorakimab was shown to effectively block the IL-33 signaling pathway. This inhibitory effect was substantiated by a reduction of more than 95% in circulating IL-33/sST2 complex levels, a decrease of more than 19% in eosinophil counts, and a 29% reduction in urinary C-C motif chemokine ligand 2 (CCL2) levels in the high-dose group compared with the placebo group (122). Furthermore, tozorakimab presented a safety and tolerability profile comparable to placebo over the 24-week intervention period (122, 123). Nevertheless, the study did not achieve its predetermined primary efficacy endpoint, as tozorakimab did not result in a statistically meaningful decrease in the urinary albumin-to-creatinine ratio (UACR) compared to placebo (122). Several interpretations of this negative result merit consideration. One possibility is that IL-33 does not play a dominant pathogenic role in the broader DKD population, despite its elevated expression in subsets of patients. Alternatively, the IL-33/ST2 pathway may contribute to disease progression primarily through the soluble decoy receptor sST2, which correlates more strongly with disease severity than IL-33 itself; selective blockade of IL-33 would not address sST2-mediated pathology. Additionally, the mandated initiation of dapagliflozin during the second half of the study may have obscured gradual effects of tozorakimab, and the 24-week follow-up may have been insufficient to capture changes in a slowly progressive disease. These findings underscore the complexity of translating preclinical observations into clinical efficacy and highlight the need for further research to identify patient subsets most likely to benefit from IL-33 pathway modulation, as well as the potential utility of targeting sST2 rather than IL-33 directly (122, 123).

Despite the fact that the initial clinical investigation in the broader DN population did not meet expectations, IL-33-targeted therapy continues to hold significant exploratory potential in specific kidney disease entities or defined patient subgroups, as supported by substantial preclinical evidence. In the context of LN, IL-33, when associated with NETs, can directly stimulate pDCs to secrete high levels of IFN-α, thereby exacerbating the autoimmune inflammatory cascade. This suggests that inhibiting the IL-33 pathway may represent an effective therapeutic strategy for LN (64). Furthermore, in animal models of IgA nephropathy, it is well established that IL-33 contributes to disease progression by promoting the expansion of ILC2s (72). This finding further expands the applicable disease spectrum of IL-33 as a therapeutic target. In addition, exploratory subgroup analysis of the FRONTIER-1 trial revealed a critical insight: a well-defined DN subgroup with elevated baseline neutrophil counts may gain greater clinical benefit from anti-IL-33 therapy (122).

**Figure 4:**
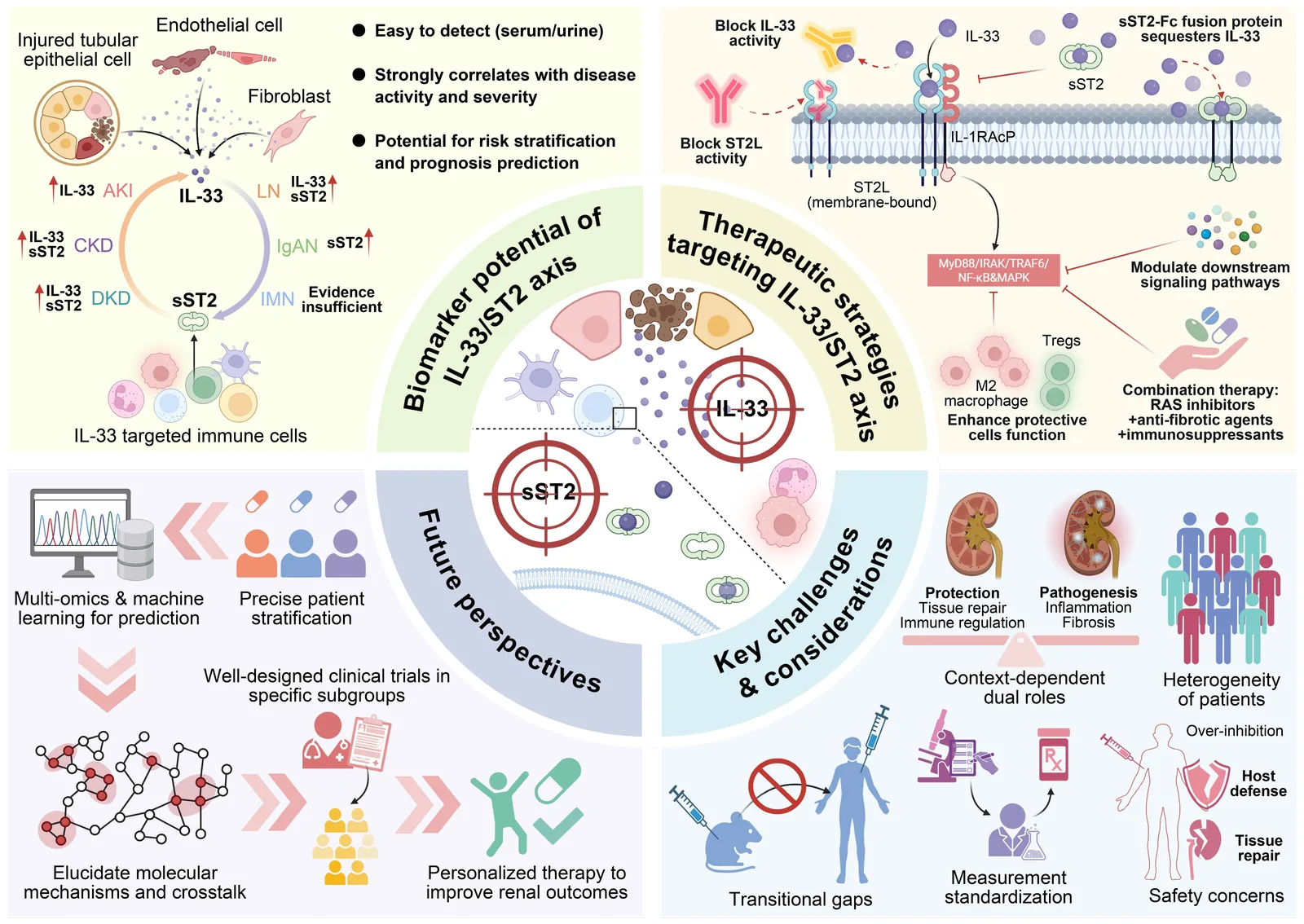
Comprehensive clinical landscape of the IL-33/ST2 axis in renal diseases. This schematic illustrates the multifaceted translational roadmap of the IL-33/ST2 axis, covering biomarker utility, therapeutic strategies, translational hurdles, and future directions. As biomarkers, both IL-33 and its soluble decoy receptor sST2, derived from injured tubular epithelial cells and immune cells, exhibit altered levels in distinct nephropathies (AKI, CKD, DKD, LN, and IgAN), highlighting their potential for non-invasive risk stratification and disease severity assessment. Therapeutically, targeting the pathway via IL-33 neutralization, ST2L blockade, or sST2-Fc fusion protein modulates downstream MyD88/NF-κB signaling, enhancing protective M2 macrophages and Tregs while suggesting combination therapies with RAS inhibitors and anti-fibrotic agents. Critical challenges include the microenvironment-dependent dual roles of IL-33 (tissue repair versus inflammation/fibrosis), patient heterogeneity, safety risks of over-inhibiting host defense, and gaps in preclinical-to-clinical translation and assay standardization. Future perspectives advocate integrating multi-omics with machine learning, enabling precise patient stratification for well-designed clinical trials, thereby elucidating molecular crosstalk and paving the way for personalized renal therapies.

When assessing the clinical application potential of IL-33-targeted therapy, researchers must take into account its inherent therapeutic risks and limitations. First, the biological functions of the IL-33/ST2 axis are highly dependent on the tissue microenvironment and disease stage, with well-documented bidirectional regulatory properties. In AKI models, endogenous IL-33 acts as an alarmin to amplify the early inflammatory response and exacerbate renal tissue damage. In contrast, exogenous low-dose IL-33 or the IL233 fusion protein exerts renoprotective effects by expanding ILC2s and Tregs, thus promoting tissue repair (29, 40, 105). This finding suggests that blocking IL-33 at an inappropriate disease stage (e.g., the acute injury phase) may disrupt the core physiological process of tissue repair. Second, studies in DN models have confirmed that global knockout of the IL-33 gene or the ST2 gene paradoxically aggravates renal pathological lesions, while only partial downregulation of this signaling pathway exerts renoprotective effects (81). This suggests that excessive and complete blockade of IL-33 signaling may disrupt its fundamental physiological function in maintaining tissue homeostasis, leading to unforeseen clinical risks. Taken together, the design of future clinical trials of IL-33-targeted agents in kidney diseases must prioritize precise patient stratification and fine-tuned dose regulation as core prerequisites, to maximize therapeutic benefits and mitigate potential risks. A comprehensive visual summary of the IL-33/ST2 axis’s biomarker potential, targeted therapeutic strategies, key clinical challenges, and future directions for precision medicine in renal diseases is provided in **Figure 4**.

## 7 Conclusion

This review addresses the contribution of the IL-33/ST2 signaling axis to renal pathophysiology. As an IL-1 family cytokine, IL-33 engages its cognate receptor ST2 to exert pleiotropic effects in kidney disorders. The IL-33/ST2 axis can promote tissue repair, mitigate inflammation, and preserve renal function, but may also exacerbate injury, drive fibrosis, and accelerate disease progression under certain conditions. This functional duality is further modulated by sST2, a decoy receptor that binds IL-33 and limits its signaling through membrane-bound ST2L. Elevated sST2 levels in CKD and diabetic nephropathy correlate more strongly with disease severity than IL-33 itself, highlighting sST2 as both a prognostic biomarker and a critical regulator of IL-33 bioavailability (80). The dynamic balance among IL-33, ST2L, and sST2, together with cellular context and disease stage, ultimately determines whether IL-33/ST2 signaling confers protection or promotes pathology (122, 123).

This review has limitations. The molecular nodes, dose dependency, spatiotemporal specificity, and dynamic switching of IL-33/ST2 signaling across acute and chronic phases require further elucidation. The regulatory role of sST2 and its interplay with IL-33 and ST2L also merit deeper investigation. Emerging factors such as gut microbiota and metabolic reprogramming, as well as cross-talk with pathways beyond TGF-β and NF-κB, remain underexplored. Future multi-omics and dynamic model studies are needed to fully delineate the regulatory network of IL-33 in kidney diseases.
